# Characteristics of banana B genome MADS-box family demonstrate their roles in fruit development, ripening, and stress

**DOI:** 10.1038/s41598-020-77870-w

**Published:** 2020-11-30

**Authors:** Yunke Zheng, Mengting Liu, Caihong Jia, Jingyi Wang, Biyu Xu, Zhiqiang Jin, Wen Li, Juhua Liu

**Affiliations:** 1 College of Horticulture, Hainan University, Haikou, 571100 China; 2 Key Laboratory of Tropical Crop Biotechnology, Ministry of Agriculture, Institute of Tropical Bioscience and Biotechnology, Chinese Academy of Tropical Agricultural Sciences, 4 Xueyuan Road, Haikou, 571101 China; 3 Hainan Academy of Tropical Agricultural Resource, Chinese Academy of Tropical Agricultural Sciences, 4 Xueyuan Road, Haikou, 571101 China

**Keywords:** Biotechnology, Molecular biology

## Abstract

MADS-box genes are critical regulators of growth and development in flowering plants. Sequencing of the *Musa balbisiana* (B) genome has provided a platform for the systematic analysis of the MADS-box gene family in the important banana ancestor *Musa balbisiana*. Seventy-seven MADS-box genes, including 18 type I and 59 type II, were strictly identified from the banana (Pisang Klutuk Wulung, PKW, 2n = 2x = 22) B genome. These genes have been preferentially placed on the banana B genome. Evolutionary analysis suggested that *M. balbisiana* MCM1-AGAMOUS-DEFICIENS-SRF (MbMADS) might be organized into the MIKC^c^, MIKC*, Mα, Mβ, and Mγ groups according to the phylogeny. MIKC^c^ was then further categorized into 10 subfamilies according to conserved motif and gene structure analyses. The well-defined MADS-box genes highlight gene birth and death in banana. *MbMADSes* originated from the same ancestor as *MaMADSes*. Transcriptome analysis in cultivated banana (ABB) revealed that *MbMADSes* were conserved and differentially expressed in several organs, in various fruit developing and ripening stages, and in stress treatments, indicating the participation of these genes in fruit development, ripening, and stress responses. Of note, SEP/AGL2 and AG, as well as other several type II MADS-box genes, including the STMADS11 and TM3/SOC1 subfamilies, indicated elevated expression throughout banana fruit development, ripening, and stress treatments, indicating their new parts in controlling fruit development and ripening. According to the co-expression network analysis, MbMADS75 interacted with bZIP and seven other transcription factors to perform its function. This systematic analysis reveals fruit development, ripening, and stress candidate *MbMADSes* genes for additional functional studies in plants, improving our understanding of the transcriptional regulation of *MbMADSes* genes and providing a base for genetic modification of MADS-mediated fruit development, ripening, and stress.

## Introduction

Bananas (*Musa* spp.) is among the oldest and most pivotal food crops. Almost all edible bananas, including diploids, triploids, and tetraploids, originate from two *Musa* ancestors: *M. acuminata* (A genome) and *M. balbisiana* (B genome)^1–3^. The genomes of these two ancestors are widely present in all *Musa* species. *Musa balbisiana* is tougher and more robust than *M. acuminata*. Hybrids possessing the B genome demonstrate increased resistance to adverse conditions^3^. Banana plants are tall (2–8 m), vigorous, suckering herbs with complex organs, including very large leaves, remarkable rhizomatous stems, and biseriately arranged flowers, which the lowernodes producing pistillate flowers and the uppermost nodes producing staminate flowers. After flowering, the ovaries of the pistillate flowers develop into fruit^3^.

The origin of this morphological complexity has been connected to the duplication of key regulatory transcription factors in MADS-box genes in plants^4,5^. According to the phylogenetic relationships, there are two categories of plant MADS-box genes, including type I and type II. The type I can be further divided into Mα, Mβ, and Mγ groups from the M domain, while the type II can be categorized into MIKC* and MIKC^C^ groups^6^. The subfamilies of MIKC^C^ MADS-box genes are exceptionally rich. Generally, 13 distinctive subfamilies recognized as AGAMOUS (AG), AGL6, AGL12, AGL15, AGL17, Bsister (GGM13), DEFICIENS/GLOBOSA (DEF/GLO), FLOWERING LOCUS C (FLC), SEPALLATA/ AGAMOUS-LIKE 2 (SEP/AGL2), APETALA1/FRUITFULL (AP1/FUL), SOLANUM TUBEROSUM MADS-BOX 11/SHORT VEGETATIVE PHASE (STMADS11 /SVP), Tomato MADS-box gene 3-like/SUPRESSOR OF OVEREXPRESSION OF CONSTANTS 1 (TM3/SOC1), and TM8 exist in MIKC^C^ MADS-box genes. In terms of the number of MADS-box genes in various organisms, to date there are 75 (37 type I and 38 type II) in rice^7^, 107 (68 type I and 39 type II) in Arabidopsis^8^, 105 (41 type I and 64 type II) in poplar^9^, 58 (20 type I and 38 type II) in grape^10^, 43 (13 type I and 30 type II) in cucumber^11^, 106 (34 type I and 72 type II) in soybean^12^ and 96 ( 31 typeI and 65 type II) in banana A genome^13^. 

MIKC^C^ MADS-box genes have pivotal parts in regulating plant growth and development, such as floral organ development and meristem determinacy^14,15^, root initiation and cell differentiation^16,17^, fruit development, ripening and quality formation^18–22^, photosynthesis and nutritional metabolism^23^, plant hormone signal transduction^24,25^. Several type I MADS-box genes participate in female gametogenesis, embryogenesis, and endosperm development^6,26,27^. Several MIKC* genes have been found to have significant roles in pollen development ^28–30^.

Though ananalysis of the MADS-box genes in the banana A genome is available^13^, a descriptive genome-wide phylogenetic and functional characterization of MADS-box genes in the banana B genome is still missing. To advance our knowledge of the characteristics of MADS-box genes in the banana B genome and to further studies on this pivotal transcription factor family, we present an in-depth analysis of the number, corresponding relationship with MaMADSes, phylogeny, location, structure, and expression and co-expression network of MADS-box genes in the recently released B genome database^31^. We find the type I and SEP/AGL2, AGL17, DEF/GLO subfamilies to be more significantly contracted and the SQUA/AP1, TM3/SOC1, and STMADS11 subfamilies to be more expanded in the B genome than the A genome. Therefore, we hypothesize that the efficient utilization and extensive sub- and neofunctionalization in these subfamilies are responsible for the extensive distribution of banana.

## Results and discussion

### Seventy-seven MADS-box genes are preferentially placed on the banana B genome

**Figure 1:**
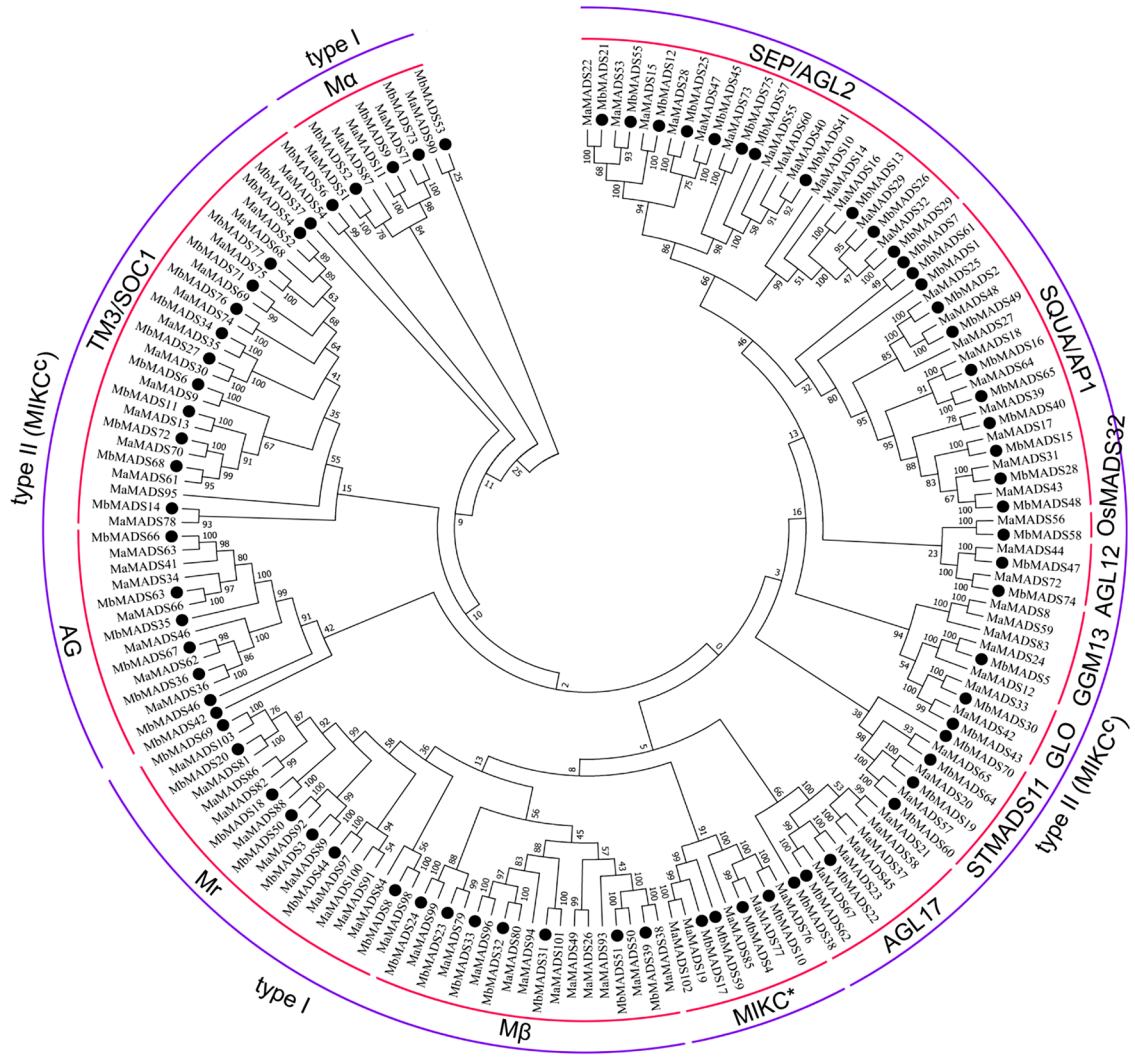
Phylogenetic analysis of the MbMADSes and MaMADSes. The Maximum Likelihood (ML) tree was drawn using MEGA 7.0 with 1000 bootstrap. The black dots indicate MbMADSes.

To strictly identify banana MADS-box genes in the B genome, we searched the banana B genome database with MADS-box sequences from the banana A genome as queries using BLAST, Hidden Markov Model searches, Swiss-Prot, and Clusters of Orthologous Genes (COG) functional annotation to establish MbMADSes. After comprehensive consideration, we identified 77 putative MADS-box members from the banana B genome. Additionally, analysis of the conserved motifs verified that the identified MbMADSes possessed the conserved MADS domain, the primary attribute of the MADS-box family. Of these 77 predicted banana MADS-box proteins, there was variation of amino acid residues that spanned 64 (MbMADS58)—818 (MbMADS32), relative molecular masses that spanned 7.4 (MbMADS58)—87.6 (MbMADS32) kDa, and isoelectric points that spanned 5.1–11.5 (Supplementary Table S1). For characterization of the evolutionary relationships among MbMADSes from the banana A and B genome, a maximum likelihood (ML) evolutionary tree was made (Fig. 1; Supplementary Table S1). Using the genome database (http://banana-genome.cirad.fr/) (released in 2019), we found that the 77 MbMADSes were localized on 11 chromosomes. The maximum number included 10 genes (13.0%) localized on chromosome 5, followed by eight (10.4%) on chromosome 1, 8, 10, and 11, seven (9.1%) on chromosomes 2, 3, and 4, and five (6.5) on chromosome 6; only four MADS-box genes were localized on chromosome 7.

### Well-defined MADS-box genes highlight gene birth and death in banana

The 77 MbMADSes were allocated to 2 groups of type I (18) and type II (59). Type I was categorized into Mα (4), Mβ (7), and Mr (7). Type II was assigned to MIKC* (4) and MIKC^c^ (55). MIKC^c^ could be assigned to 10 subfamilies, which is one less than MaMADSes because of the missed DEF. Subfamily OsMADS32-like only contains one MbMADS protein, while subfamily TM3/SOC1 has the maximum number (13) of MbMADSes members, then 11, 11, 7, 4, 3, 2, 2, and 1 members for subfamilies SEP/AGL2, SQUA/AP1, AG, STMADS11, AGL17, GLO, AGL12, and GGM13, respectively.

Due to gene duplication-transposition, the gene birth and death rate for type I MADS-box is higher than type II^32^. Most type I genes are functionally redundant or silent and only partly required for regulating coenocytic development, while their other functions remain elusive^33,34^. In comparison with MaMADSes, the number of type I MbMADSes was greatly decreased, with 13 fewer than MaMADSes^13^. This result suggests that type I MADS-box in the B genome shows a higher gene death rate and that the B genome banana efficiently uses type I to regulate female gametogenesis and seed development, which is consistent with the report that the banana B genome has greater gene family contraction and loss than the A genome^31^.

 The number of type II MbMADSes was relatively stable, with only six less than MaMADSes. The subfamilies of SQUA/AP1, TM3/SOC1, and STMADS11 displayed slightly greater gene expansion, with two, two, and one more than in the A genome. Subfamilies SEP/AGL2, AGL17, DEF/GLO, GGM13, and MIKC* displayed slight gene contraction with three, three, three, one, and one less than the A genome. Nevertheless, AG, AGL12, and OsMADS32-like remained unchanged. These results indicate that type II MbMADSes were more conserved than type I, and mild sub- and neofunctionalization in these subfamilies may be linked to the complex morphology and environmental distribution of banana.

 Being similar to MaMADSes, the MIKC^c^ subfamilies, such as STK and FLC, are exclusive to the banana B genome (Fig. 1). FLC subfamily genes determine flowering time^35^. The cause for the death of the FLC subfamily might be consistent with the tropical character of banana, the flowering of which does not need low temperature stimulation, allowing the plant to flower randomly at any time. STK subfamily genes control ovule development^36^. The death of STK corroborates the greatly decreased number of developing ovules and the key evolutionary step of the long-term selection for seedless fruits in wild banana, which caused sterility and improved the palatability of wild seedy banana fruits^37^ During banana development, 20–70% of ovules are lost. Even in cases where the ovules appear normal, approximately 50% of the normal ovules remain unfertilized despite a sufficient supply of pollen to the stigma^38^. The remaining developing ovules might be controlled by other subfamilies such as AG, SQUA/AP1, and TM3/SOC1. Together, this highlights that banana can realize its evolutionary advantages and fully utilize its MADS-box genes in flower and fruit development.

### Conserved and variable structure exhibit adaptability

The structure of a MADS-box gene decides its function. Generally, MADS-box genes consist of four domains: MADS, I, K, and C. The I domain may be responsible for protein dimer formation. The K domain is responsible for protein dimerization, and the C terminal domain may be responsible for transcriptional activation and protein complex formation^39,40^.

**Figure 2:**
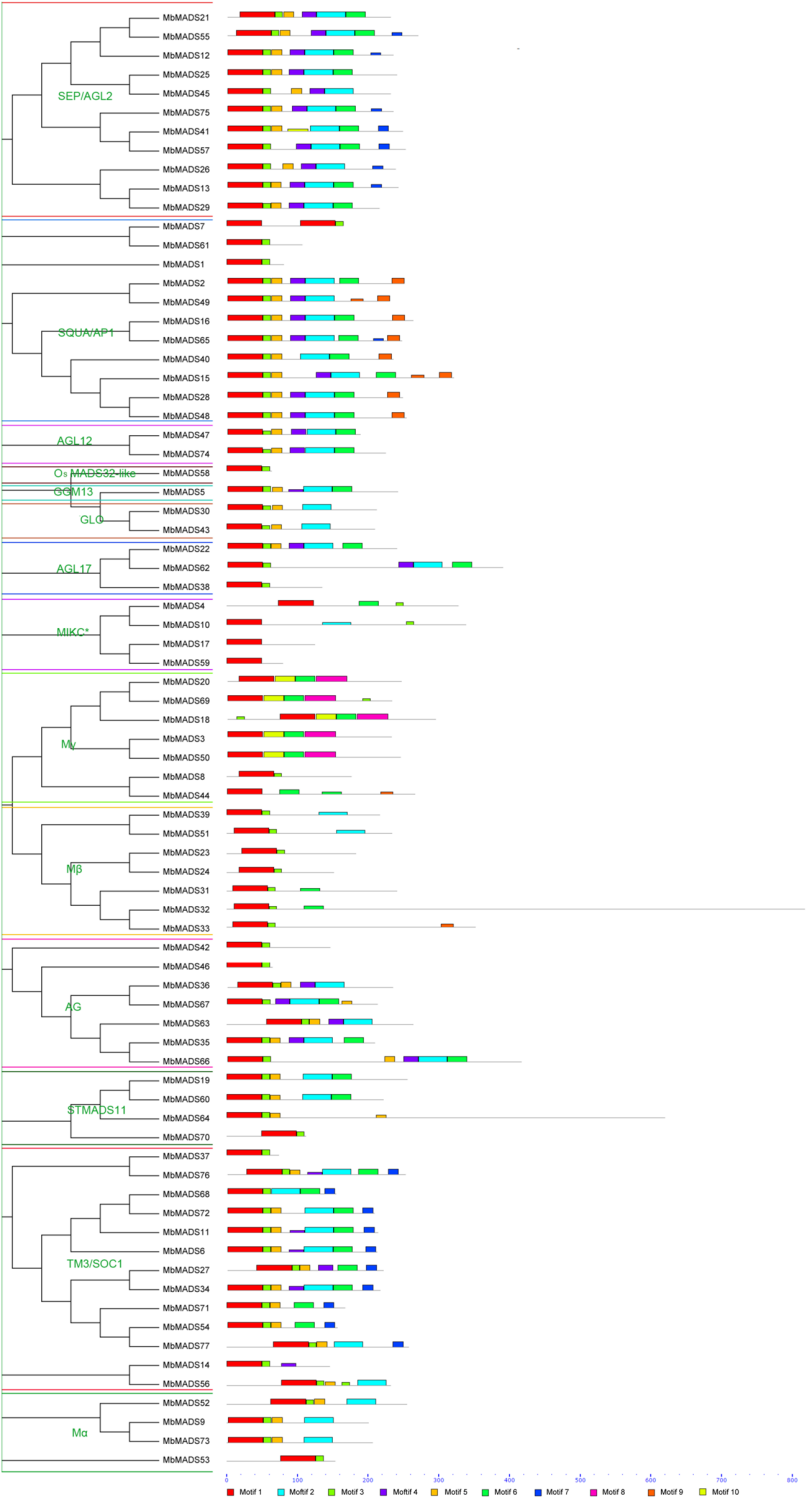
Conserved motif analyses of MbMADSes. All motifs were identified by MEME database with the complete amino acid sequences of MbMADSes. The classification of MbMADSes were shown based on the phylogenetic relationship.

**Figure 3:**
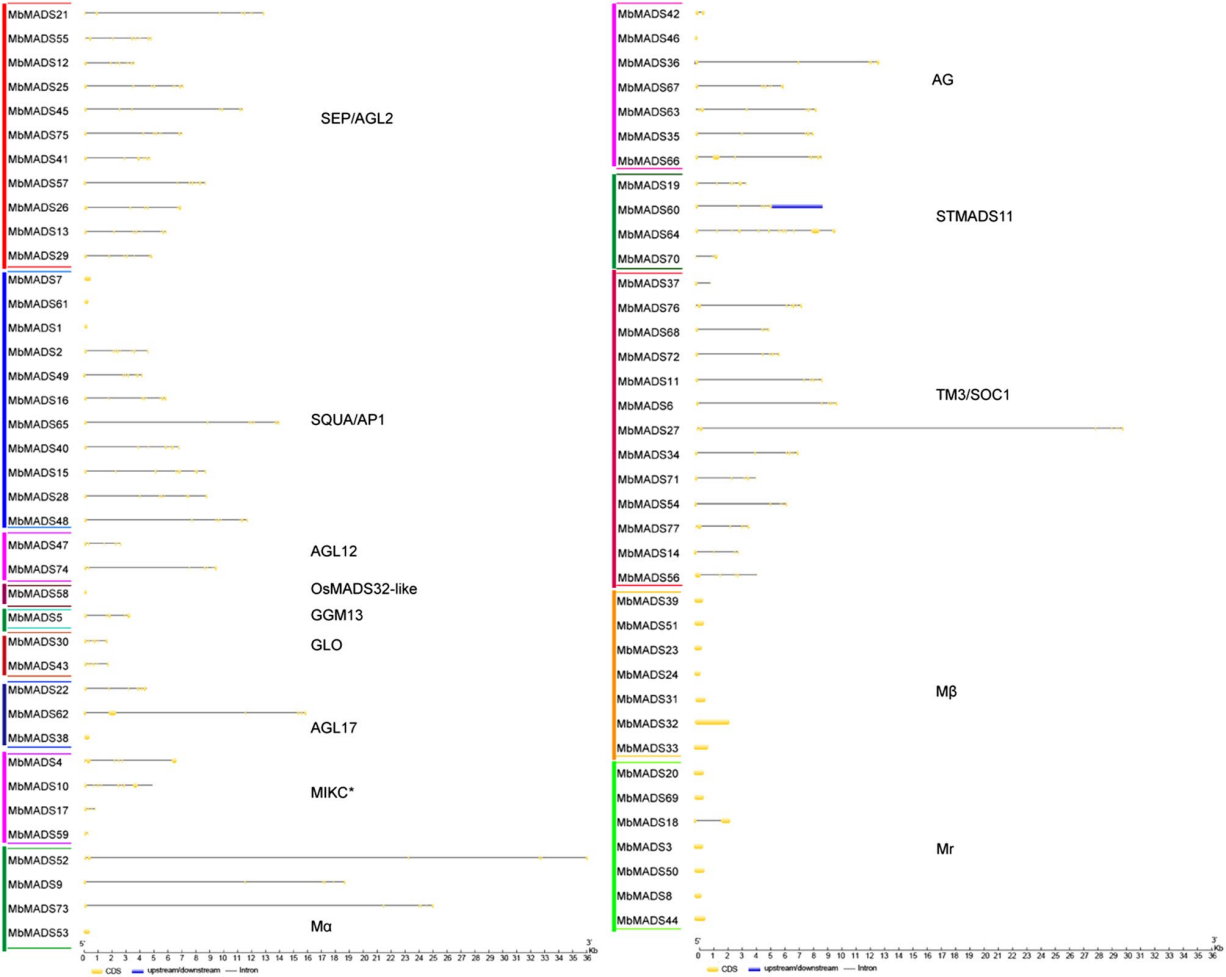
Gene structure analyses of *MbMADSes*. Exon–intron structure analyses were performed by GSDS database. The blue boxes indicate upstream/downstream, the yellow boxes indicate exons, and the black lines indicate introns.

 To obtain the characteristics of the MbMADSes proteins, we used MEME software to identify 10 conserved motifs in total, and we used the InterPro database to annotate them (Fig. 2). Moreover, the exon–intron structure was also obtained by gene structure display server (GSDS) (http://gsds.cbi.pku.edu.cn/). All the MbMADSes proteins contain the conserved MADS domain (Motif 1). In terms of both the conserved domain and exon–intron analysis, type I MbMADSes were the most simple MADS-box proteins and contained two to five motifs. Fifteen out of 18 type I MbMADSes, except MbMADS 9, 52, and 73, contained a MADS domain (Motif 1) and a variable C terminal domain (Motif 7, 8, 9 or 10). Consistent with the domain analysis, 14 out of 18 type I MbMADSes, except MbMADS 9, 18, 52, and 73, were intronless (Fig. 3). This simple structure might facilitate their role in evolution and the regulation of seed development^33,34^.

 Compared with type I, the structure of type II MbMADSes was more complex, and most contained six to seven motifs. Ninety-three percent (55/59) of type II MbMADSes, except for MIKC*, contained Motif 3 with or without Motif 5, which was annotated as I domain; 69% (41/59) of type II MbMADSes, except for all MIKC*, three SQUA/AP1 subfamily MbMADS1, 7, and 61, one OsMADS32-like subfamily MbMADS58, one AGL17 subfamily MbMADS38, two AG subfamily MbMADS42 and 46, two STMADS11 subfamily MbMADS64 and 70, and five TM3/SOC1 subfamily MbMADS14, 27, 37, 54, and 71, contained Motif 2 with or without Motif 4 or 6 and were annotated as the basic K domain. Most SEP/AGL2, SQUA/AP1, and TM3/SOC1 subfamily members contained Motif 7 with or without Motif 8, 9, and 10 and were annotated as another important C domain.Conversely, 90% of type II MbMADSes, except for three SQUA/AP1 subfamily members MbMADS1, 7, and 61, one OsMADS32-like subfamily MbMADS58, one AGL17 subfamily MbMADS38, and one AG subfamily MbMADS46, contained one or more exons and introns. The variable number of coding exons and multiple exon–intron structures suggest that type II MbMADSes contribute greatly to the high adaptability of bananas containing the B genome and experience less selection pressure than type I MbMADSes, which is consistent with the report of Hoffmeier et al. (2018)^41^.

### *MbMADSes* originate from the same ancestor with *MaM*ADSes

**Figure 4:**
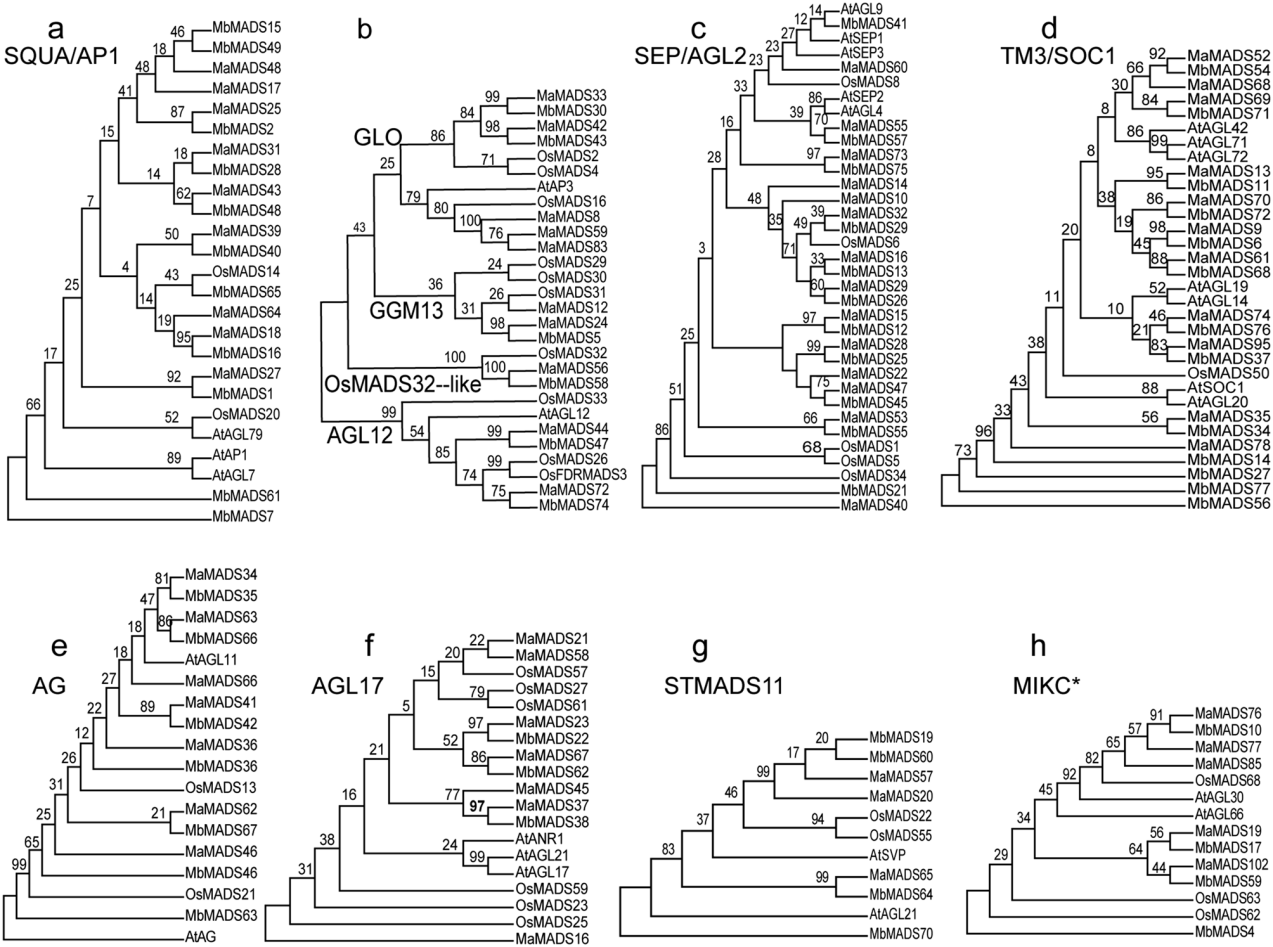
Phylogeny patterns of type II MADS-box gene families between *M. acuminata* (A-genome) and *M. balbisiana* (B-genome). (**a**) SQUA/AP1; (**b**) GLO, GGM13, OsMADS32-like and AGL12; (**c**) SEP/AGL2; (**d**) TM3/SOC1; (**e**) AG; (**f**) AGL17; (**g**) STMADS11; (**h**) MIKC*.

**Figure 5:**
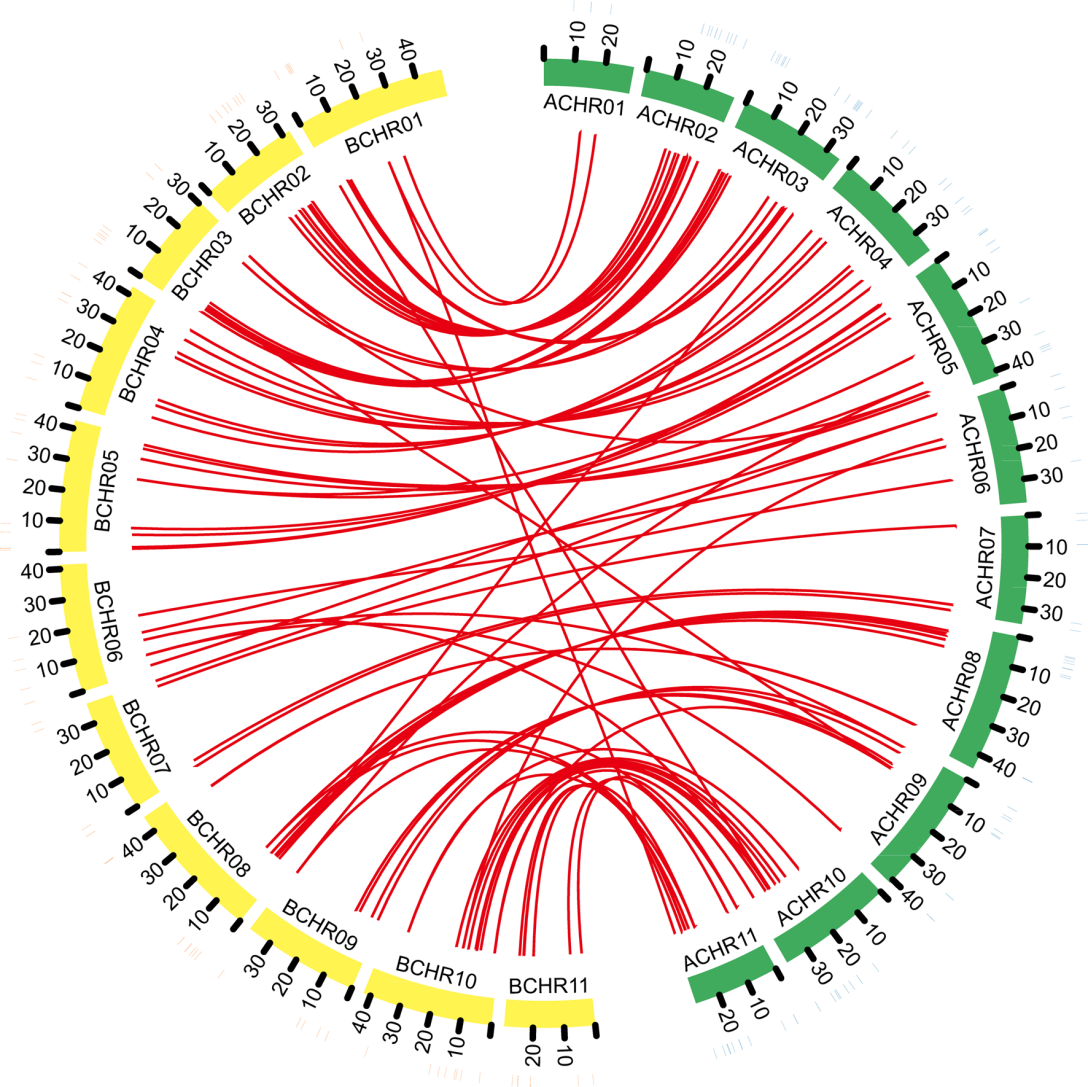
Syntenic patterns of MADS-box genes between *M. acuminata* (A-genome) and *M. balbisiana* (B-genome). ACHR01-11, 11 chromosomes of *M. acuminata*; BCHR01-11, 11 chromosomes of *M. balbisiana*.

To investigate the evolutionary position of *MbMADSes,* 11 ML phylogenetic trees of MADS-box genes from *Arabidopsis thaliana*, rice (*Oryza sativa*), and the banana A and B genome were constructed, as shown in Figs. 1, 4, and 5. The result showed that all banana MADS-box genes, except for the TM3/SOC1 and STMADS11 subfamilies, are linked more tightly with rice than *A. thaliana.* The cause of this close relationship may be that banana and rice are monocotyledons. Furthermore, a lot of closely related orthologous MADSes, such as MbMADS65 with OsMADS14 (Fig. 4a), MaMADS8, 59, and 83 with OsMADS16 (Fig. 4b), and MaMADS26, 29, 13, 14, MbMADS32, 29, 16 with OsMADS6 (Fig. 4c), exist in banana and rice, suggesting that ancestral MADSes genes were present before banana and rice diverged (Fig. 4).

Synteny was observed inthe A and B genome divergence (Fig. 1; Supplementary Table S2). For example, MaMADS81 and 103 are sister to MbMADS20 and 69, respectively. A total of 73 pairwise genes were formed between the banana A and B genome in Fig. 1, among which 65 pairwise genes were formed by MbMADSes with MaMADSes. All MbMADSes could locate their counterparts in the A genome (Fig. 5; Supplementary Table S2). A one-to-one correspondence was generated, with a high frequency of 75% on the same chromosome and 25% on the different chromosomes. For example, MbMADS6 and MaMADS9 are sisters located on the same chromosome 1, while MbMADS2 and MaMADS25 are sisters located on chromosomes 1 and 3, respectively (Fig. 5; Supplementary Table S2). This close evolutionary relationship suggests that A and B genome banana originated from the same ancestor and a number of syntenic events occurred in these lineages, leading to the syntenic divergence of banana A and B before the polyploidization of banana (Fig. 1). The one-to-one correspondence from the different chromosomes indicates that chromosomal cross over, exchange, recombination, as well as transposable elements and long terminal repeat retrotransposons might have occurred during divergence from the common ancestor^42,43^. This result corroborates the report that irregularities, including bridges, fragments, and lagging univalents, can be detected in a significant proportion of clones during microsporogenesis and the second meiotic division^3^. Seven pairwise genes were formed by MaMADSes themselves, and one pairwise gene was formed by MbMADS7 and MbMADS61. The close gene vicinity to each other suggests that subfamily expansion may have proceeded via tandem duplications (Fig. 1).

### Conserved and differential expression profiles of *MbMADSes* genes in Banana (ABB) 

**Figure 6:**
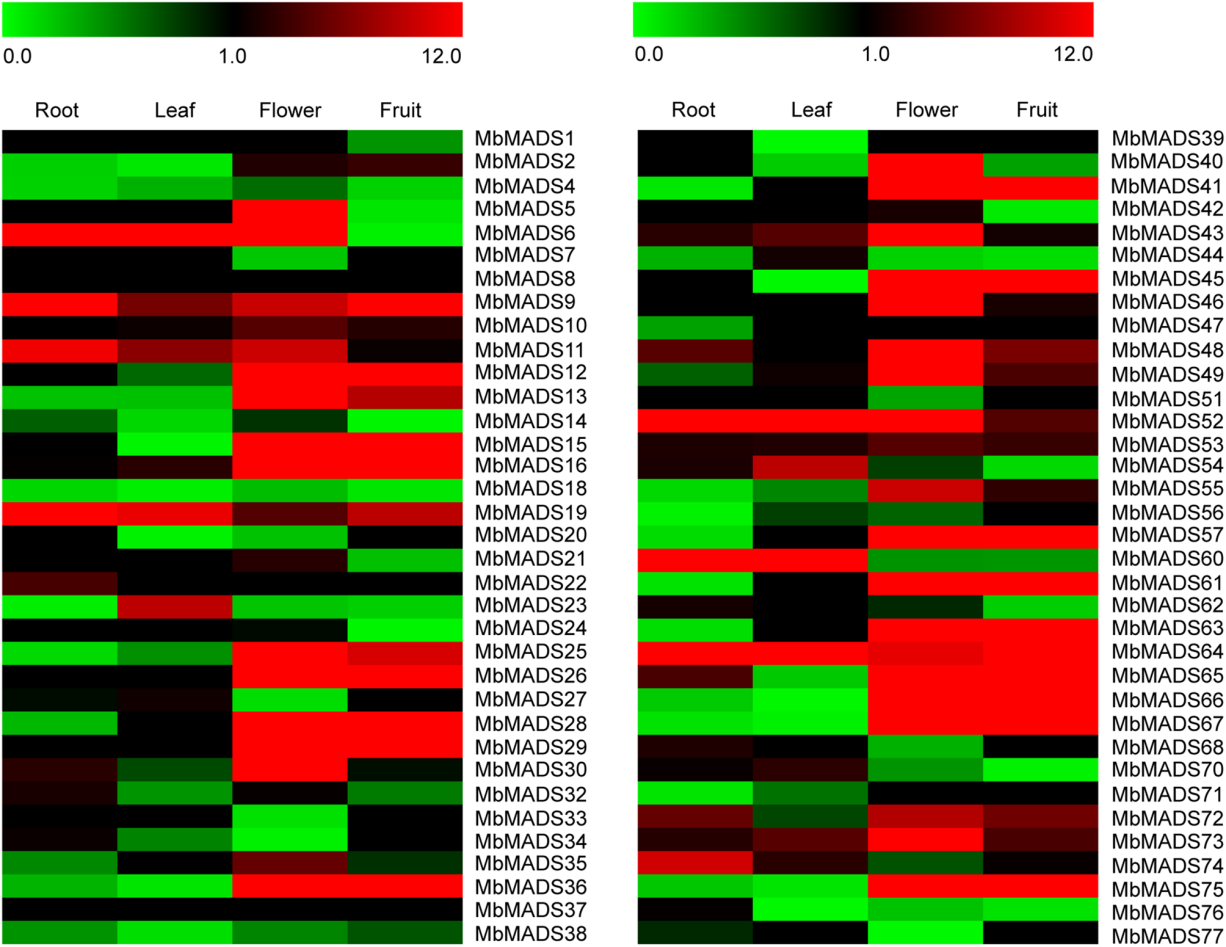
Expression patterns of *MbMADSes* in the roots (R), leaves (L), flowers (Fl) and fruits (Fr) of banana (ABB). The heat map with clustering was created based on the FPKM value of *MbMADSes*. Differences in gene expression changes are shown in color as the scale.

To evaluate the organ-specific expression characteristics of MADS genes in banana (ABB), the roots, leaves, flowers, and fruits were subjected to RNA-seq analysis. Of the 77 *MbMADSes* genes, 68 genes (except for *MbMADS3, 8, 17, 31, 37 50, 58, 59, 69*) were expressed in at least 1 examined organ (Fig. 6; Supplementary Table S3). Fifty-one *MbMADSes* (75.0%) demonstrated expression in the roots, 45 (66.2%) in the leaves, 63 (92.6%) in the flowers, and 56 (82.4%) in the fruits, of which eight (15.7%), five (11.1%), 29 (46.0%), and 19 (33.9%) demonstrated high expression levels (value > 10) in the roots, leaves, flowers, and fruits, respectively, and 0 (0%), one (0%), three (10.3%), and three (15.8%) genes displayed significantly elevated expression levels (value > 100) in the roots, leaves, flowers, and fruits, respectively. Moreover, the expression values of *MbMADS36* (AG subfamily) in the flowers, *57* (SEP/AGL2 subfamily) in the fruits, and *75* (SEP/AGL2 subfamily) in the flowers and fruits were greater than 200. The most highly expressed genes in the roots, leaves, flowers, and fruits were *MbMADS60* (STMADS11 subfamily), *MbMADS6* (TM3/SOC1 subfamily), *MbMADS36* (AG subfamily), and *MbMADS75* (SEP/AGL2 subfamily), which reached 40, 150, 206, and 319, respectively. These findings demonstrated that MADS-box genes were conserved and divergently expressed in various banana organs. The finding that so many *MbMADSes* were highly expressed in the roots is in agreement with the review that MADS-box genes are fundamental in root development^44^. The highly elevated expression levels and gene numbers in the flowers and fruits imply that *MbMADSes* have more significant parts in the flowers and fruits than in other organs, which is in line with the early study that MADS-box transcription factors are pivotal in flower and fruit development^45,46^.

**Figure 7:**
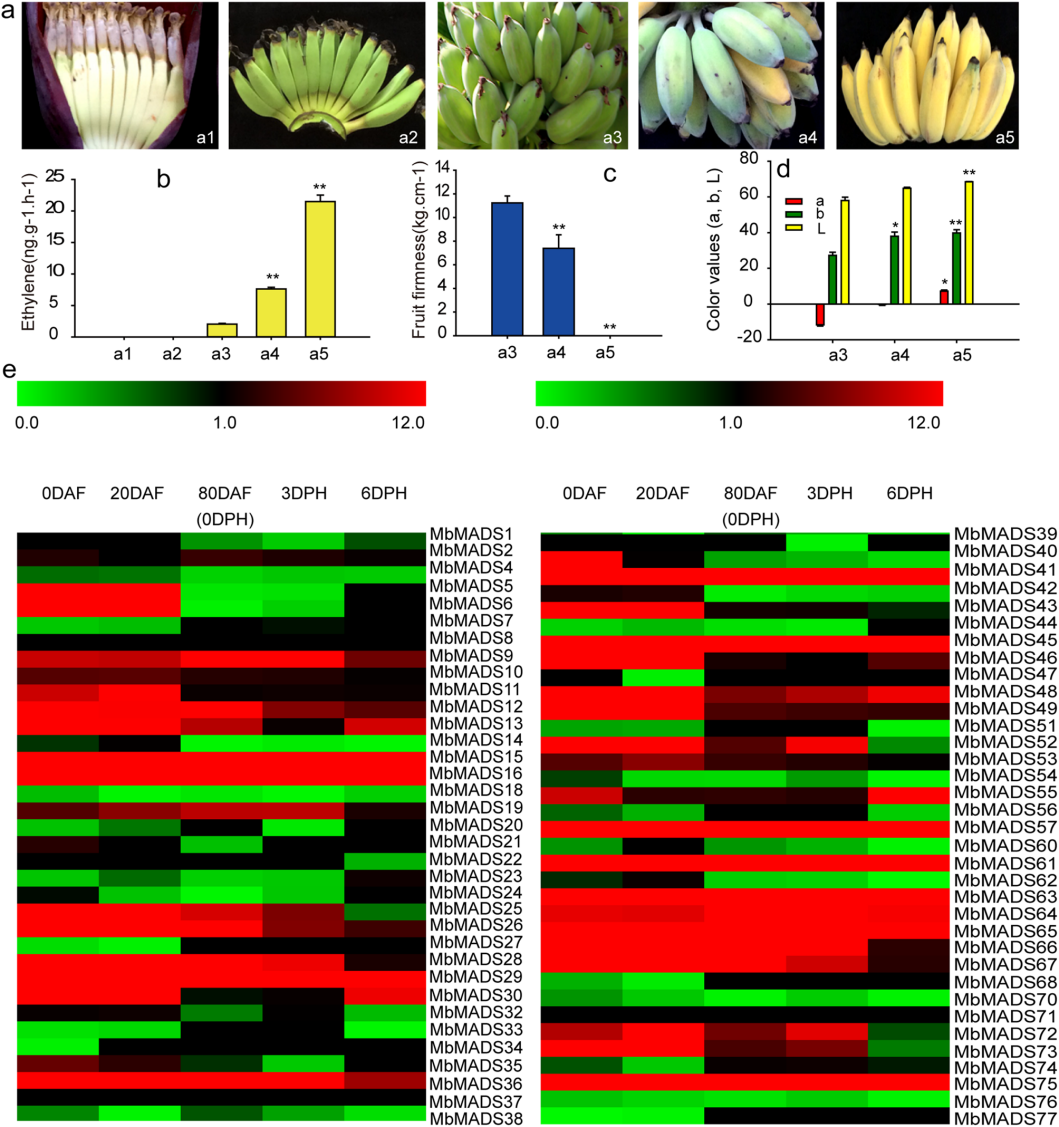
Phenotypes, fruit ripening qualities and expression patterns of *MbMADSes* in different stages of fruit development and ripening in banana (ABB). (**a**) Phenotypes of different stages of fruit development and ripening in banana (ABB). a1–a5 represent 0 DAF, 20 DAF, 80 DAF (0 DPH), 3 DPH and 6 DPH, respectively; (**b**) Ethylene release during fruit development and ripening process; (**c**) Fruit pulp firmness during fruit ripening process; (**d**) Fruit peel color (a, b, and L) during fruit ripening process; (e) The heat map with clustering was created based on the FPKM value of *MbMADSes*. Differences in gene expression changes are shown in color as the scale. The statistical significance of the differences was assessed by ANOVA (*p < 0.05; **p < 0.01).

 The phenotypes of fruit development and ripening process were as shown in Fig. 7a. Along with fruit ripening, the ripening-related physiological parameters significantly changed. The ethylene production significantly increased and reached the highest of 21.5 ng. g^−1^ h^−1^ while the fruit pulp firmness greatly decreased and reached the lowest of 0 at 6 DPH (Fig. 7b,c). Moreover, the colors of a, b, and L gradually increased and reached the highest of 7.2, 39.8 and 68.5 at 6 DPH, respectively (Fig. 7d). These results were consistent with our recently report of Wang et al. (2019)^31^.

To evaluate the contribution of *MbMADSes* genes in fruit development and ripening, the expression of *MbMADSes* genes was evaluated in fruits sampled from 0, 20, and 80 days after flowering (DAF) and 0, 3, and 6 days postharvest (DPH) of the fruits (Fig. 7e; Supplementary Table S4). Among the 77 *MbMADSes*, 68 genes (except for *MbMADS3, 8, 17, 31, 37 50, 58, 59, 69*) were differentially expressed at various fruit development and ripening stages. Sixty-three (92.6%), 61 (89.7%), 56 (82.4%), 57 (83.8%), and 53 (77.9%) *MbMADSes* were expressed at 0 DAF, 20 DAF, 80 DAF (0 DPH), 3 DPH, and 6 DPH, of which 29 (46.0%), 30 (49.1%), 19 (33.9%), 17 (29.8%), and 15 (28.3%) genes, respectively, were highly expressed (value > 10) at each stage, and among which four (13.7%), four (13.3%), four (21.1%), three (17.6%), and three (20.0%) genes displayed super expression levels (value > 100). Furthermore, the most highly expressed genes at 0 DAF, 20 DAF, 80 DAF (0 DPH), 3 DPH, and 6 DPH were *MbMADS36* (AG), 3*6* (AG), *75* (SEP/AGL2), *75* (SEP/AGL2), and *75* (SEP/AGL2), respectively, which reached 206, 408, 319, 279, and 1174, respectively; this suggest that these genes might function prominently in developmental and ripening processes of banana fruit. These results were closely aligned with the report that the AG and SEP subfamilies are the key regulators of fruit development and ripening^21,22,47^. The finding that *MbMADS36* was highly expressed in both flower and fruit development could be explained by the morphologically in distinguishable pistillate and staminate flowers that are biseriately arranged in a cluster^3^.

### Expression profiles of *MbMADSes* genes under abiotic and biotic stresses in Banana (ABB) 

Banana is a valuable fruit of tropic and subtropic environments and can adapt to environmental stresses^48,49^. The prolonged process of banana evolution represents the long history of plant domestication. Banana propagates vegetatively by divisions known as “pups” or “suckers.” As the scale of production increased, the sterile cultivars were grown in close proximity in large quantities, resulting in attack by pathogens because of a lack of genetic diversity^50^. *Fusarium oxysporum* f. sp. *cubense* tropical race 4 (Foc TR4) is believed to be a major and destructive disease of banana, ranking in the top six of significant global plant diseases^51^. Foc TR4 targets banana plant roots and colonizes the vascular system of the rhizome and pseudostem. Within 5–6 months of planting, distinctive internal and external wilting symptoms can typically be observed^52^. Thus, understanding the molecular mechanism of abiotic stress and Foc TR4 infection is a priority for the sustainable development of the banana industry.

**Figure 8:**
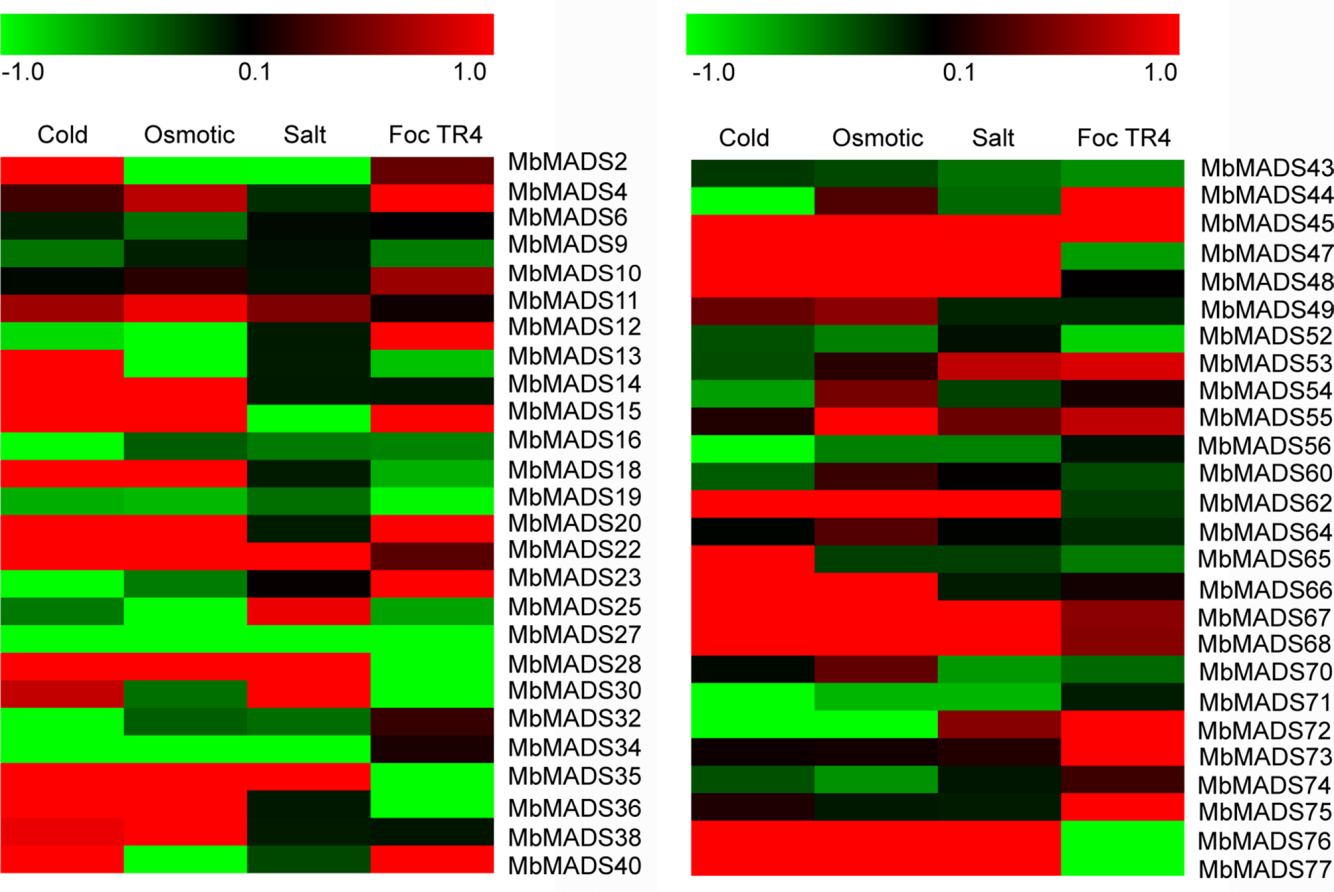
Expression patterns of *MbMADSes* in response to cold, osmotic, salt and Foc TR4 treatments in banana (ABB). Log_2_ based FPKM value was used to create the heat map. Differences in gene expression changes are shown in color as the scale.

 To evaluate the stress-response expression profiles of *MbMADSes*, the leaves were sampled under cold, osmotic, salt treatments, and the roots infected with Foc TR4 for RNA-seq analysis (Fig. 8; Supplementary Table S5). A total of 52 genes (except for 25 *MbMADSes*, including *MbMADS1*, *3*, *5*, *7*, *8*, *17*, *21*, *24*, *26*, *29*, *31*, *33*, *37*, *39*, *41*, *42*, *46*, *50*, *51*, *57*, *58*, *59*, *61*, *63*, and *69*) showed transcriptional changes after abiotic stress and Foc TR4 treatments. Eighteen (34.6%), 17 (32.7%), 18 (34.6%), and 23 (44.2%) genes showed up-regulation, while 20 (38.5%), 22 (42.3%), 26 (50%), and 24 (46.2%) genes showed down-regulation under cold, osmotic, salt, and Foc TR4 infection, respectively. Furthermore, seven, six, two, and six *MbMADSes* genes were significantly up-regulated (value > 1), and nine, seven, four, and seven genes were significantly down-regulated (value < − 1) under cold, osmotic, salt, and Foc TR4 treatments, respectively. Based on this, it is evident that a greater number of genes were significantly regulated by cold, osmotic, salt stress, and Foc TR4 infection. Additionally, *MbMADS20* (Mr), *67* (AG), *76* (TM3/SOC1), and *23* (Mβ) showed significant up-regulation, and *MbMADS71* (TM3/SOC1), *25* (SEP/AGL2), *27* (TM3/SOC1) and *77* (TM3/SOC1) showed significantly down-regulation under cold, osmotic, salt stress, and Foc TR4 treatments, respectively.

 MADS-box genes in plants are well-known for their functions in different significant processes throughout growth and development, especially in flower development^47^. However, further study indicated that the expression of certain MADS-box genes is influenced by abiotic stresses,including salt, drought, osmotic, heat, and cold stress^7,53–58^.

 In this report, 52 genes responded to cold, osmotic, salt, and Foc TR4 treatments, suggesting that these genes have played a significant role in domestication. This result is consistent with other reports that AGL17 clade genes are predominantly expressed in the roots^59–61^. SOC1, which has well-detailed parts in reproductive transition and carpel development, has been demonstrated to participate in the formation of periodic lateral roots^62^. *XAANTAL1* (*XAL1*; AGL12) regulates the auxin-dependent cell cycle, which affects root growth and flowering time^17^. Generally, the characterization of the function of the different type I genes is poor, but it has been reported that several type I genes participate in female gametogenesis, embryogenesis, and seed development^5,6,26,27^. Here, type I MADS-box genes, such as *MbMADS20* (Mr) and *MbMADS23* (Mβ), as well as *MbMADS67*(AG), *MbMADS76* (TM3/SOC1), *MbMADS71* (TM3/SOC1), and *MbMADS25* (SEP/AGL2), were induced by cold, osmotic, salt stress, and Foc TR4, suggesting their new function in environmental adaptation; an aspect that requires additional investigation.

### Interaction network of preferentially expressed gene and validation 

**Figure 9:**
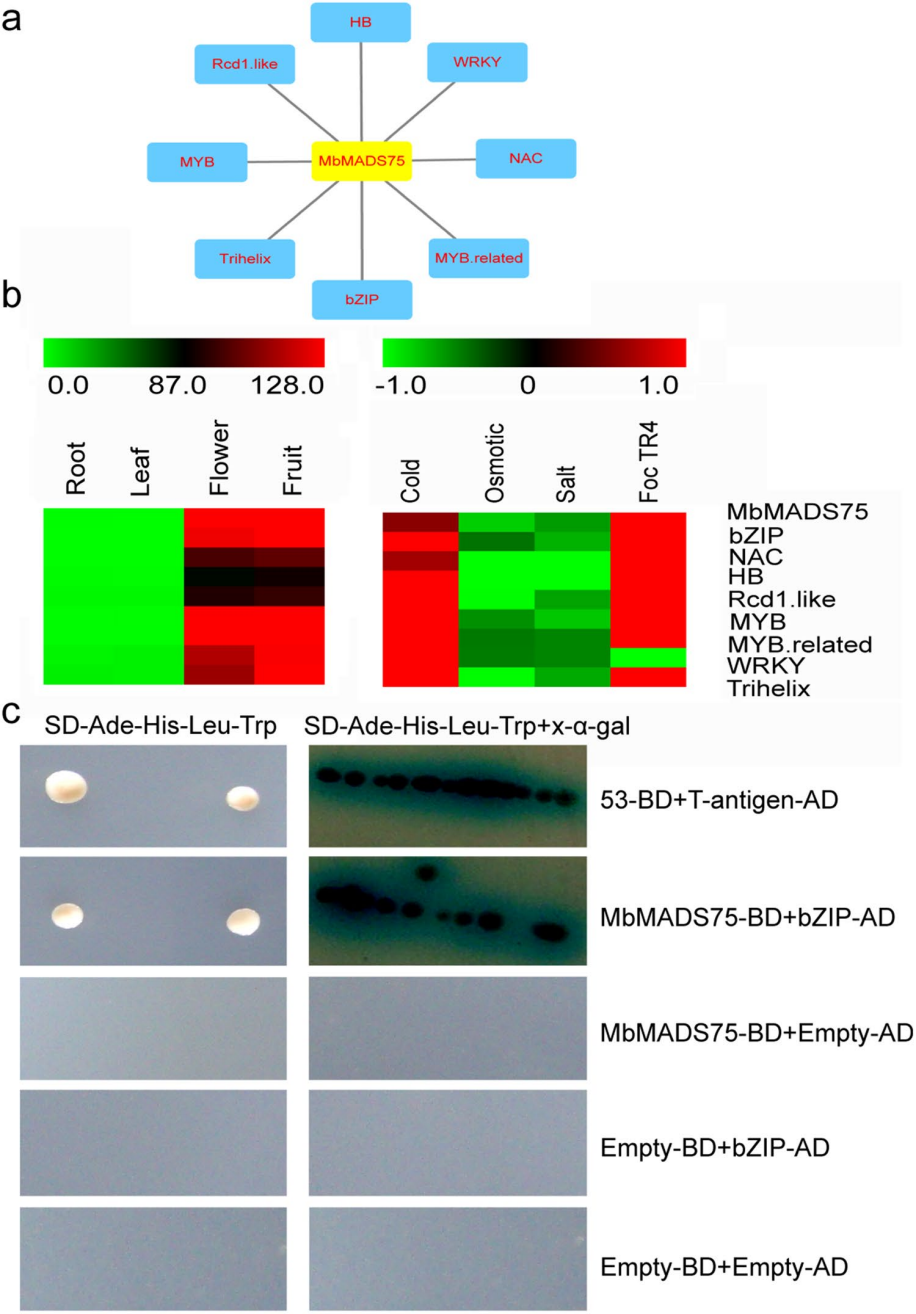
Interacted network of MbMADS75 using Cytoscape and validation by qRT-PCR and Y2H. (**a**) co-expression network. (**b**) Identification of interacted proteins by qRT-PCR. (**c**) Identification of interaction of MbMADS75 and bZIP by Y2H.

Protein–protein interactions are vital for MADS-boxprotein function; hence, evaluating the interaction networks is valuable for characterizing gene function mechanisms. Herein, *MbMADS75*, which exhibits high expression during fruit development and in the ripening stage, was chosen to evaluate possible protein interaction and co-expression networks with Cytoscape software^63^, assisting further studies relating to their biological function obtained from interactions validated by experiment. One MbMADS75-mediated network was built and eight interactive proteins for MbMADS75 were obtained (Fig. 9a and Supplementary Table S6). MbMADS75 interacted with basic region/leucine zipper motif (bZIP, Mb_10_t14180.1), WWRKYGQK (WRKY, Mb_04_t18050.1), myeloblastosis (MYB, Mb_07_t05690.1), MYB-related (Mb_04_t26670.1), NAM, ATAF1/2 and CUC2 (NAC, Mb_11_t15670.1), homeobox (HB, Mb_04_t35340.1), the radical-induced cell death protein 1-like (Rcd1.like, Mb_01_t28400.1), and helix–loop–helix–loop–helix (Trihelix, Mb_04_ t32090.1), indicating that these transcription factors interacted with MbMADS75 to play key roles during banana fruit growth and processes for ripening.

 These eight interacted transcription factors were validated by quantitative real-time (Fig. 9b). Moreover, MbMADS75 and bZIP (Mb_10_t14180.1) were selected to identify their interaction by Yeast Two-Hybrid assay (Fig. 9c). The reason for this selection is that bZIP transcription factors are crucially implicated in plant development and responses to numerous stresses^64,65^. The results of the qRT-PCR demonstrated that the eight interacting genes possessed the same expression pattern as *MbMADS75*, except that the WRKY was down-regulated by Foc TR4, which requires further investigation. The result of the Yeast Two-Hybrid assay demonstrated that MbMADS75 could interact with bZIP (Mb_10_t14180.1) to perform its function.

In conclusion, we identified a total of 77 MADS-box genes from the banana (Pisang Klutuk Wulung, PKW) B genome. We classified these as the MIKC^c^, MIKC*, Mα, Mβ, and Mγ groups according to the phylogeny, and also organized MIKC^c^ into 10 subfamilies. The well-defined *MbMADSes* highlight gene birth and death in banana. *MbMADSes* originate from the same ancestor as *MaMADSes*. Major genes that demonstrated high expression in fruit development, ripening, and the stress treatments were part of the SEP/AGL2 and AG subfamilies. Several type I and other subfamilies, including the TM3/SOC1 and STMADS11 *MbMADSes* genes, demonstrated high expression in the process of banana fruit growth, ripening, and stresses, which suggests their novel parts in controlling fruit development, ripening, and stress responses. Interactive network analysis indicated that MbMADS75 interacted with bZIP and seven other transcription factors to perform its functions. These findings contribute greatly to our understanding of the contributions of *MbMADSes* in the regulation of banana fruit development, ripening, and environmental adaptation processes, and enable further breeding and genetic improvements in agriculture.

## Methods

### Plant materials and treatments 

We acquired various stages of developing bananas fruits, namely, 0 DAF, 20 DAF, and 80 DAF (0 DPH), from the banana plantation of the Institute of Tropical Bioscience and Biotechnology (Chengmai, Hainan, 20N, 110E). Postharvest banana hands at comparable developmental stages were chosen and permitted to naturally ripen. Bananas were examined simultaneously for ethylene release, pulp firmness and color (‘a’, ‘b’ and ‘L’ value) during fruit ripening period. The 3 DPH and 6 DPH fruits were obtained according to the ethylene production. We obtained young banana seedlings at the five-leaf stage from the Banana Tissue Culture Center (Danzhou, Institute of Bananas and Plantains, Chinese Academy of Tropical Agricultural Sciences) and cultivated them in soil under greenhouse conditions of 28 °C, 70% relative humidity, and 200 μ mol m^−2^ s^−1^ light intensity with a 16 h light/8 h dark cycle. The roots and leaves at the five-leaf stage, flowers at 0 DAF, and fruits at 80 DAF were sampled for organ-specific gene expression analysis. For fruit development and ripening process gene expression analysis, fruit pulp tissues of 0 DAF, 20 DAF, 80 DAF, 3 DPH, and 6 DPH were collected. For osmotic and salt stress treatments, five-leaf stage banana seedlings grown in soil were sprayed with 200 mM mannitol or 300 mM NaCl for 7 d. Banana seedlings were subjected to 4 °C for 22 h for the cold stress treatment. The biotic stress treatment was according to Wang et al. (2012)^66^. Five-leaf stage banana seedling roots were saturated in a Foc TR4 spore suspension of 1.5 × 10^6^ conidia/mL, with the whole root system collected at 0 and 2 days post-infection (DPI). All samples were flash-frozen in liquid nitrogen and stored at − 80 °C until total RNA extraction for the transcriptomic assay.

### Ethylene production, fruit firmness and peel color analyses

Fruit ethylene production was measured according to the method of Liu et al. (2015)^21^. Fruit firmness was measured according to the method of Li et al. (2013)^67^. The instrumental measurement of banana peel color was carried out according to the method of Jaiswal et al. (2014)^68^. At least three biological replicates were assessed, and all of the data were analyzed using One-way analysis of variance (ANOVA) and Student’s *t*-tests for determination of significant differences.

### Identification and evolutionary analyses 

The whole MADS-box protein sequences of the banana A genome, banana B genome, *Arabidopsis*, and rice were acquired from the Banana Genome Hub released January 2016 and October 2019 (http://banana-genome-hub.southgreen.fr/download)^31^, RGAP (http://rice.plantbiology.msu.edu/) and TAIR (http://www.arabidopsis.org/) databases, respectively. To identify the banana B genome MADS-box family genes, local Hidden Markov Model-based searches (http://hmmer.wustl.edu/) was first conducted based on known MADS-box to explore the banana genome database^69^; Following this, we carried out BLAST searches to establish the anticipated MbMADSes in the banana database, using all *Arabidopsis* and rice MADSes as queries. We ultimately assessed each of the candidate protein sequences using the CDD (http://www.ncbi.nlm.nih.gov/cdd/) and PFAM (http://pfam.sanger.ac.uk/) databases. Then, we used multiple sequence alignments to verify the conserved domains of the predicted MbMADSes proteins. Further, we used Clustal X 2.0 to perform sequence alignments of the full-length MADSes proteins from banana, *Arabidopsis,* and rice. A maximum likelihood (ML) evolutionary tree with 1000 bootstrap replicates was produced in MEGA 7.0 software to assess the phylogenetic relationships^70^.

### Protein characteristics and sequence analyses

The molecular weight and isoelectric points of the predicted MbMADSes proteins were predicted with the ExPASy proteomics server (http://expasy.org/). Using the MEME program (http://meme.nbcr.net/meme/cgi-bin/meme.cgi), we identified the conserved motifs in the full-length banana MADS proteins based on the parameters: maximum motif number of 10 and optimum motif width of between 6 and 50. We subsequently annotated all identified motifs using InterProScan (http://www.ebi.ac.uk/Tools/pfa/iprscan/). We identified the gene structures of banana MbMADSes using the GSDS program.

### Transcriptome analysis

The samples of different organs, different treatments, and various development and ripening stages of bananas, namely, 0 DAF, 20 DAF, 80 DAF (0 DPH), 3 DPH, and 6 DPH, were gathered to extract total RNA utilizing the plant RNeasy extraction kit (TIANGEN, Beijing, China) for transcriptome analysis. Three μg of total RNA from each sample was converted to cDNA using a RevertAid First-Strand cDNA Synthesis Kit (Fermentas, Beijing, China). cDNA libraries were constructed using TruSeq RNA Library Preparation Kit v2, and were subsequently sequenced on the Illumina HiSeq 2000 platform using the Illumina RNA-seq protocol^31^. Each sample had two replicates. The average sequencing depth was 5.34X. Paired end reads with 90-bp were produced on HiSeq 2000 platform of all samples. A total of 159.14 Gb of high-quality clean data was produced and aligned using SOAPaligner/SOAP2 version 2.21 with parameters “-m 0 -x 1000 -s 40 -l 32 -v 5 -r 1 -p 3”^71^. Adapter sequences in the raw sequence reads were extracted using the FASTX-toolkit. Following sequence quality assessment and removal of low-quality sequences by FastQC, we obtained clean reads. We then mapped these clean reads to the DH-PKW genome (*Musa balbisiana*, B-genome, 2n = 22)^31^ using Tophat v.2.0.10. Transcriptome assembly was conducted in Cufflinks^72^. We calculated the gene expression levels as Reads Per Kilobase of exon model per Million mapped reads (FPKM). We determined differentially expressed genes with DEGseq^73^. RNAseq reads was deposited in NCBI-SRA database (accession number: PRJNA343716).

### Quantitative real-time PCR analysis

The changes in the transcriptome of *MbMADS75* and the other eight interacted genes were evaluated by qRT-PCR analysis on Stratagene Mx3000P Real-Time PCR system with SYBR Premix Ex Taq (TaKaRa, Japan). The PCR amplification conditions utilized for each of the reactions were as follows: 10 min at 95 °C, followed by 40 cycles of 10 s at 95 °C, 15 s at 50 °C and 30 s at 72 °C. Target gene relative expression levels were estimated using the 2–^ΔΔ^Ct method^74^. The reaction specificities for every primer pair were evaluated utilizing qRT-PCR melting curve analysis, agarose gel electrophoresis, and sequencing of the PCR products (Supplementary Table S7). *MaRPS2* (HQ853246) and *MaUBQ2* (HQ853254) constituted the internal controls that were used to normalize the target gene expression^75^. Each treatment sample had a corresponding regularly-watered control, with three independent biological replications for each sample. We sampled treatment and control plants at every time point for expression analysis, and contrasted the relative expression levels of genes in every treatment time point with those in every time point under normal conditions.

### Regulatory network construction

Based on the B genome database^32^ and transcriptome analysis, we selected MbMADS75—which was especially expressed throughout the fruit developing and the ripening process—as the “from node” and the interactive proteins as “to node direction” to establish a gene regulatory network diagram using Cytoscape software (version 3.4.0).

### Y2H assay

*MbMADS75* was cloned into pGBKT7 to fuse with the bait domain (BD) with the primers P1: 5′-CCGAATTCATGGGGAGGGGGAGGGTGG-3′ and P2: 5′-CCGTCGACTTCCAGCCA TGCAGGCAT-3′. The PCR products were digested with *Eco*RI and *Sal*I and cloned into the *Eco*RI-*Sal*I site of the pGBKT7 bait vector. *bZIP1* was cloned into pGADT7 to fuse with the activation domain (AD) of GAL4 using the primers (P1: 5′-CCGAATTCATGGACCCTGC CGGGCCGC-3′, P2: 5′-CCGAGCTCCATAAAGTCCATTGGATC-3′). Selective synthetic dropout medium plates (SD/-Trp, SD/-Trp/-His, SD/-Trp/-His + X-α-gal) were used to assay self-activation. The combinations were then concurrently transformed into the yeast strain AH109 based on the protocol. Transformants possessing the plasmids *MbMADS75-*BD with pGADT7 and pGBKT7 with *bZIP*-AD were selected as negative controls. The interactions were assessed based on growth on selective medium (SD/-Ade/-His/-Leu/-Trp + x-α-gal) in accordance with the Clontech protocol (http://www.clontech.com/).

## Supplementary information

Supplementary Tables.
